# Trends in acute myocardial infarction mortality in the United States, 1999–2023

**DOI:** 10.3389/fmed.2026.1817513

**Published:** 2026-05-19

**Authors:** Jiayin Zhu, Yinhui Pei

**Affiliations:** 1 Department of Clinical Medicine, North China University of Science and Technology, Tangshan, China; 2 Department of Immunology, School of Elementary Medicine, North China University of Science and Technology, Tangshan, China

**Keywords:** acute myocardial infarction, mortality trends, CDC WONDER, age-adjusted mortality rate, health disparities

## Abstract

**Background:**

Although mortality from acute myocardial infarction (AMI) has declined historically, the burden remains substantial. This study evaluated long-term temporal trends and population-level disparities in AMI-related mortality among US adults aged ≥ 45 years from 1999 to 2023.

**Methods:**

Mortality data were retrieved from the CDC WONDER underlying cause of death (UCD) and Multiple Cause of Death (MCD) datasets. We identified death records listing AMI (ICD-10: I21–I22) as the cause of death. Crude mortality rates and age-adjusted mortality rates (AAMRs) per 100,000 population were calculated, with standardization to the 2000 US population. Joinpoint regression models were employed to estimate the annual percent change (APC) and average annual percent change (AAPC). To ensure robustness, a sensitivity analysis was conducted using the MCD dataset, defining AMI as a contributing cause of death with cardiovascular disease (ICD-10: I00–I99) as an underlying cause of death.

**Results:**

A total of 3,255,707 AMI-related deaths were analyzed. The overall AAMR decreased from 206.12 (95% CI: 205.21–207.03) in 1999 to 60.71 (95% CI: 60.31–61.11) in 2023, with an overall AAPC of −4.94%. Joinpoint analysis identified decline during 1999–2018, followed by a plateau during 2018–2021 (APC: −0.02%) and a subsequent accelerated decline during 2021–2023 (APC: −8.74%). While male AAMRs remained consistently higher than female AAMRs (81.86 vs. 43.12 in 2023), females exhibited a faster rate of decline (AAPC: −5.30% vs. −4.87%). Racial disparities persisted, with non-Hispanic Black individuals showing higher mortality than non-Hispanic White individuals. Urban-rural analysis (1999–2020) revealed that rural areas faced the highest mortality burden and the slowest decline (AAPC: −3.92%) compared to large metropolitan areas (AAPC: −5.58%). Sensitivity analyses yielded results consistent with the primary findings.

**Conclusion:**

Despite a significant long-term reduction in AMI-related mortality in the United States, progress has been uneven, characterized by a phase-specific plateau and persistent geographic and racial disparities. Targeted public health strategies and resource allocation are essential to address the high burden in rural areas and among vulnerable populations to further reduce health inequities.

## 1 Introduction

AMI is one of the core clinical phenotypes of ischemic heart disease leading to premature death and disability, and its event adjudication and classification should follow unified epidemiological and clinical standards. The Fourth Universal Definition of Myocardial Infarction emphasizes diagnosis based on the combination of evidence of myocardial injury and ischemia, and distinguishes different etiologic types, thereby providing a methodological basis for comparing outcomes across periods and regions (1). Over the past several decades, mortality from coronary heart disease/ischemic heart disease in the United States has shown an overall downward trend, which prior studies have attributed to risk-factor control (e.g., reduced smoking prevalence and improved lipid and blood pressure management) and broader uptake of evidence-based therapies (2).

However, recent national analyses suggest a slowing in the decline of cardiovascular/metabolic mortality, with signals of stagnation or rebound even in some age groups (3). Among young adults, the decline in coronary heart disease mortality may also have “stalled,” suggesting that changes in population risk profiles and healthcare accessibility may influence long-term trends (4). In addition, marked urban-rural disparities exist in the United States: cardiovascular mortality is higher in rural areas and has improved less, suggesting that imbalances in healthcare resources, emergency care networks, and social determinants may continue to affect outcomes (5). During the COVID-19 pandemic, cardiovascular mortality in the United States increased in phases and showed pronounced interstate differences, providing important context for interpreting recent trend fluctuations (6).

The CDC WONDER database provides a standardized data source for long-term, nationwide, and stratified trend assessments. It allows comprehensive and convenient access to relevant US data and includes county-level mortality data and multidimensional demographic variables (7). AMI is a critical manifestation of cardiovascular disease, with coronary atherosclerosis as its pathological basis; plaque rupture induces thrombosis, leading to acute vascular occlusion and myocardial ischemic necrosis. The occurrence of MI (myocardial necrosis) can further impair cardiac function and may trigger more severe cardiovascular diseases such as heart failure and arrhythmia, creating a vicious cycle. Therefore, when evaluating overall trends with AMI as the contributing cause of death, conducting sensitivity analyses using cardiovascular disease as an underlying cause of death may help verify the robustness of the conclusions.

Based on national mortality registry data from CDC WONDER, this study used Joinpoint regression to quantify long-term trends, inflection points, and stratified differences in AMI-related AAMRs among US adults aged ≥ 45 years from 1999 to 2023, and characterized spatial heterogeneity at the state level to identify demographic differences (including sex, race, urbanization level, and geographic region), thereby providing evidence for targeted interventions and public health policies aimed at optimizing clinical management (8).

## 2 Materials and methods

### 2.1 Study background and population

This descriptive study used death certificate data provided through the US Centers for Disease Control and Prevention (CDC) WONDER platform to analyze mortality trends related to AMI. For our primary analysis, we used the underlying cause of death (UCD) dataset, defining AMI mortality as ICD-10 I21–I22 listed as the underlying cause of death. The primary objective was to evaluate the distribution of AMI-related mortality in the UCD dataset among individuals aged 45 years and older from 1999 to 2023; this dataset contains death certificate data from all 50 US states and the District of Columbia (9). Given the mandatory nature of death registration in the US, the proportion of missing data for key demographic variables (age, sex, and state) was negligible (< 0.1%); cases with missing information were excluded, as this threshold is unlikely to introduce significant selection bias. Underlying cause of death (UCD) refers to the disease or injury that initiated the chain of events leading directly to death, which is recorded in Part I of the death certificate. Contributing Cause of Death refers to conditions that aggravated or contributed to death but did not initiate the fatal pathological chain, which can be recorded in lower lines of Part I or Part II of the death certificate. To address potential information bias arising from variations in physician coding practices, for sensitivity analysis, we used the Multiple Cause of Death (MCD) public dataset (10). In these analyses, AMI-related mortality was defined by the presence of ICD-10 codes I21–I22 in any of the 20 possible cause-of-death fields (including Part I or Part II of the death certificate), that is, AMI was defined as a contributing cause while a broader cardiovascular disease (ICD-10 codes I00–I99) was designated as the underlying cause of death. Notably, the sensitivity analysis yielded results that were numerically higher in absolute AAMRs but meaningfully consistent in terms of temporal trends, identified joinpoints, and the magnitude of APCs, confirming that the observed patterns are robust and independent of the primary versus secondary coding hierarchy. Because the analysis was based solely on de-identified federal data, institutional review board (IRB) review was not required. By adhering to the STROBE guidelines, this study maintained methodological transparency regarding data limitations and bias mitigation strategies.

### 2.2 Data extraction

Mortality was stratified by major sociodemographic and geographic factors, including sex, race/ethnic background, level of urbanization, and US Census-designated region (11). Racial and ethnic groups included Hispanic or Latino, non-Hispanic (NH) White, and non-Hispanic (NH) Black or African American individuals, consistent with classification conventions commonly used in earlier studies based on the CDC WONDER. Urban-rural classification was based on the 2013 NCHS Urban-Rural Classification Scheme (12). Therefore, this study was limited to evaluating urbanization trends from 1999 to 2020. According to US Census Bureau standards, the country was divided into four geographic regions–Northeast, Midwest, South, and West. Sex was further categorized as male and female.

### 2.3 Statistical analysis

To analyze the nationwide distribution of AMI-related mortality from 1999 to 2023, we calculated crude mortality rates (CDR) and age-adjusted mortality rates (AAMRs) per 100,000 population with 95% confidence intervals (CIs), stratified by year, sex, race/ethnicity, state, and urban-rural status. CRs were calculated by dividing the annual number of AMI-related deaths by the corresponding US population, while AAMRs were directly standardized to the 2000 US standard population. Temporal trends were evaluated using the National Cancer Institute Joinpoint Regression Program (version 5.1.0). We opted for Joinpoint regression over alternative models, such as generalized additive models (GAMs) or segmented regression with predefined knots, because it utilizes a permutation-based approach to objectively identify the number and location of statistically significant inflection points, thereby minimizing researcher bias. Furthermore, this log-linear approach provides a parsimonious interpretation of mortality “phases” through the annual percent change (APC) and average annual percent change (AAPC)—standardized metrics in public health that offer clearer linear summaries of trends for policymakers than complex smoothed splines. Parallelism tests were performed to determine whether time trends differed significantly across demographic subgroups. Statistical significance was set at *P* < 0.05, determined by a two-tailed *t*-test evaluating whether the slope differed significantly from zero.

## 3 Results

### 3.1 Overall AMI-related mortality trends

**FIGURE 1:**
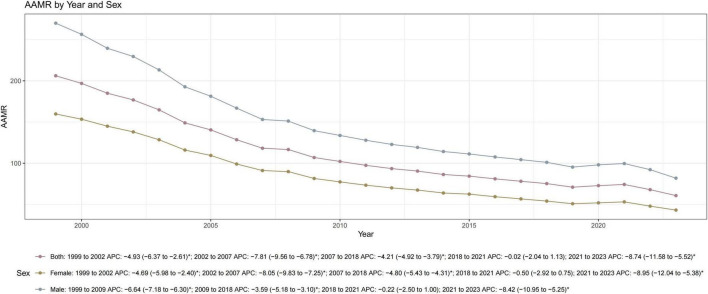
Trends in AMI-related age-adjusted mortality rates (AAMRs) by sex among US adults aged ≥ 45 years, 1999–2023. AMI-related deaths were identified using ICD-10 codes I21–I22. AAMRs are standardized to the 2,000 US standard population and expressed per 100,000 population. Joinpoint regression identified declining trends in both sexes, with men consistently having higher AAMRs than women. Women exhibited a faster overall decline (AAPC: –5.30) compared to men (AAPC: –4.87). APC, annual percent change; AAPC, average annual percent change. The asterisk (*) indicates that the APC was significantly (*P* < 0.05).

From 1999 to 2023, a total of 3,255,707 AMI-related deaths were recorded among US adults aged ≥ 45 years (Table 1 and Supplementary Table 1). The overall age-adjusted mortality rate (AAMR) declined substantially from 206.12 per 100,000 in 1999 to 60.71 in 2023, yielding an overall average annual percent change (AAPC) of −4.94, *p* < 0.05. Joinpoint regression identified five distinct temporal phases. Four declining Joinpoint segments were statistically significant (*p* < 0.05), whereas the 2018–2021 plateau was not statistically significant (APC = −0.02%, *p* = 0.80). Specifically, a steady decline from 1999 to 2007, a continued but slightly slower decline through 2018, a notable plateau during the pandemic period (2018–2021; APC = −0.02, *p* > 0.05), and a renewed, accelerated decline from 2021 to 2023 (Figure 1). Sensitivity analyses using the Multiple Cause of Death (MCD) dataset confirmed these trends, identifying the same 2018–2021 plateau (APC = −0.02, *P* = 0.80) and subsequent decline (Supplementary Figure 1), ensuring the robustness of the primary underlying cause of death (UCD) findings.

**TABLE 1:** Frequency and age-adjusted mortality rates per 100,000 for AMI-related deaths among US adults aged 45 years and older, stratified by sex, race/ethnicity, census region, and urbanization level.

Characteristic	Subgroup	Deaths	Population	AAMR 1999 (95% CI)	AAMR 2023 (95% CI)	AAPC (95% CI)
Overall		3,255,707	3,043,725,672	206.12 (205.21–207.03)	60.71 (60.31–61.11)	−4.94 (−5.17 to −4.75)
Gender	Male	1,786,967	1,427,661,266	269.92 (268.22–271.62)	81.86 (81.16–82.56)	−4.87 (−5.08 to −4.72)
	Female	1,468,740	1,616,064,406	159.75 (158.73–160.78)	43.12 (42.67–43.57)	−5.30 (−5.56 to −5.10)
Race	Hispanic	175,601	308,938,513	162.46 (158.51–166.42)	45.95 (44.84–47.07)	−5.11 (−5.37 to −4.84)
	NH White	2,655,431	2,245,334,172	207.72 (206.72–208.72)	64.25 (63.77–64.74)	−4.63 (−4.82 to −4.47)
	NH Black or African American	336,332	322,117,448	235.65 (232.22–239.08)	69.52 (68.14–70.90)	−4.99 (−5.20 to −4.81)
Census region of United States	Northeast	604,309	574,121,780	198.87 (196.92–200.81)	49.93 (49.10–50.77)	−5.39 (−5.57 to −5.24)
	Midwest	791,828	668,417,547	220.53 (218.61–222.46)	66.89 (65.97–67.80)	−4.65 (−4.83 to −4.50)
	South	1,312,860	1,127,663,361	225.10 (223.49–226.72)	68.99 (68.30–69.67)	−4.81 (−5.02 to −4.62)
	West	546,710	673,522,984	162.62 (160.79–164.45)	49.81 (49.05–50.57)	−4.65 (−4.88 to −4.43)
**Urbanization**		**Deaths**	**Population**	**AAMR 1999 (95% CI)**	**AAMR 2020 (95% CI)**	**AAPC (95% CI)**
	Large	1,322,751	1,393,581,246	192.21 (190.97–193.46)	59.44 (58.89–59.99)	−5.58 (−5.70 to −5.45)
	Medium/small	901,172	794,661,897	197.88 (196.26–199.50)	73.43 (72.64–74.23)	−4.81 (−4.98 to −4.62)
	Rural/counties	732,212	434,230,530	258.25 (255.90–260.60)	116.41 (115.01–117.82)	−3.92 (−4.12 to −3.72)

### 3.2 Sex-specific trends

Mortality decreased progressively in both sexes, though men consistently maintained higher AAMRs than women throughout the study period (81.86 vs. 43.12 in 2023). However, the pace of improvement was more rapid in women, who exhibited a steeper overall decline (AAPC: −5.30) compared to men (AAPC: −4.87). Both demographic groups experienced the 2018–2021 plateau before trends resumed their downward trajectory (Figure 1 and Table 1).

### 3.3 Race and ethnicity differences

**FIGURE 2:**
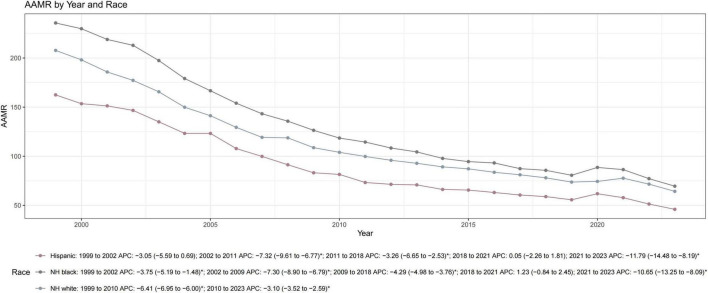
Trends in AMI-related age-adjusted mortality rates (AAMRs) by race and ethnicity among US adults aged ≥ 45 years, 1999–2023. Data are presented for three major groups: non-Hispanic (NH) White, NH Black/African American, and Hispanic/Latino. While all groups experienced significant long-term reductions, NH Black individuals consistently maintained the highest mortality burden throughout the study period. The Hispanic/Latino population showed the most pronounced overall decline (AAPC: –5.11). NH, non-Hispanic; APC, annual percent change; AAPC, average annual percent change. The asterisk (*) indicates that the APC was significantly (*P* < 0.05).

Persistent disparities were observed across the three major racial and ethnic groups (Table 1 and Supplementary Table 2). Non-Hispanic Black individuals consistently exhibited the highest AAMRs, followed by non-Hispanic White and Hispanic/Latino individuals. While all groups saw substantial reductions, the Hispanic/Latino population experienced the most pronounced overall decline (AAPC: −5.11), whereas non-Hispanic White individuals showed the slowest rate of improvement (AAPC: −4.63; Figure 2).

### 3.4 Geographic and urban-rural gradients

**FIGURE 3:**
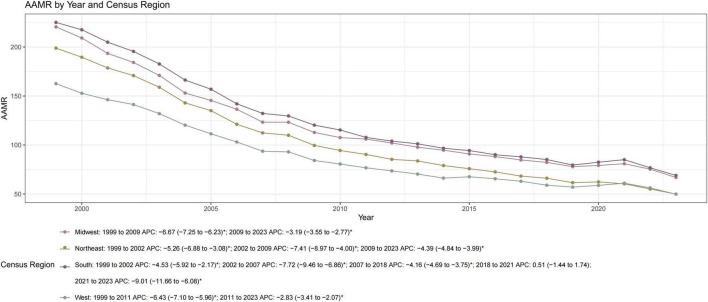
Trends in AMI-related age-adjusted mortality rates (AAMRs) by US Census Region among adults aged ≥ 45 years, 1999–2023. Trends are stratified into the Northeast, Midwest, South, and West regions. The South consistently recorded the highest AAMRs, while the Northeast achieved the largest overall decline (AAPC: –5.39, *P* < 0.05). Parallelism tests confirmed significant differences in trend slopes across regions (*P* < 0.05). APC, annual percent change; AAPC, average annual percent change; CI, confidence interval. The asterisk (*) indicates that the APC was significantly (*P* < 0.05).

**FIGURE 4:**
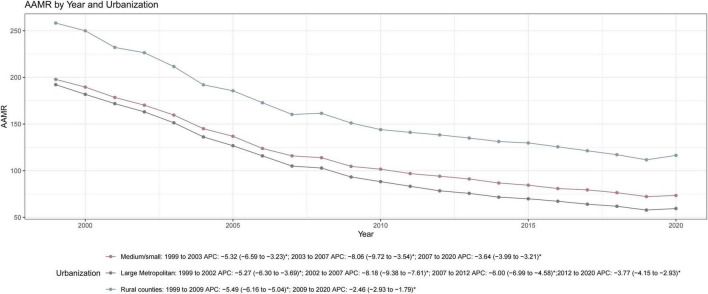
Trends in AMI-related age-adjusted mortality rates (AAMRs) by urbanization level among US adults aged ≥ 45 years, 1999–2020. Urbanization levels are classified according to the 2013 NCHS Urban-Rural Classification Scheme. Analysis is limited to 2020 due to classification comparability. Rural/county areas consistently faced the highest mortality burden and the slowest rate of decline (AAPC: –3.92, *P* < 0.05), while large metropolitan areas showed the most significant improvement (AAPC: –5.58, *P* < 0.05). Parallelism tests confirmed significant differences in trend slopes across urbanization levels (*P* < 0.05). APC, annual percent change; AAPC, average annual percent change; CI, confidence interval; NCHS, National Center for Health Statistics. The asterisk (*) indicates that the APC was significantly (*P* < 0.05).

**FIGURE 5:**
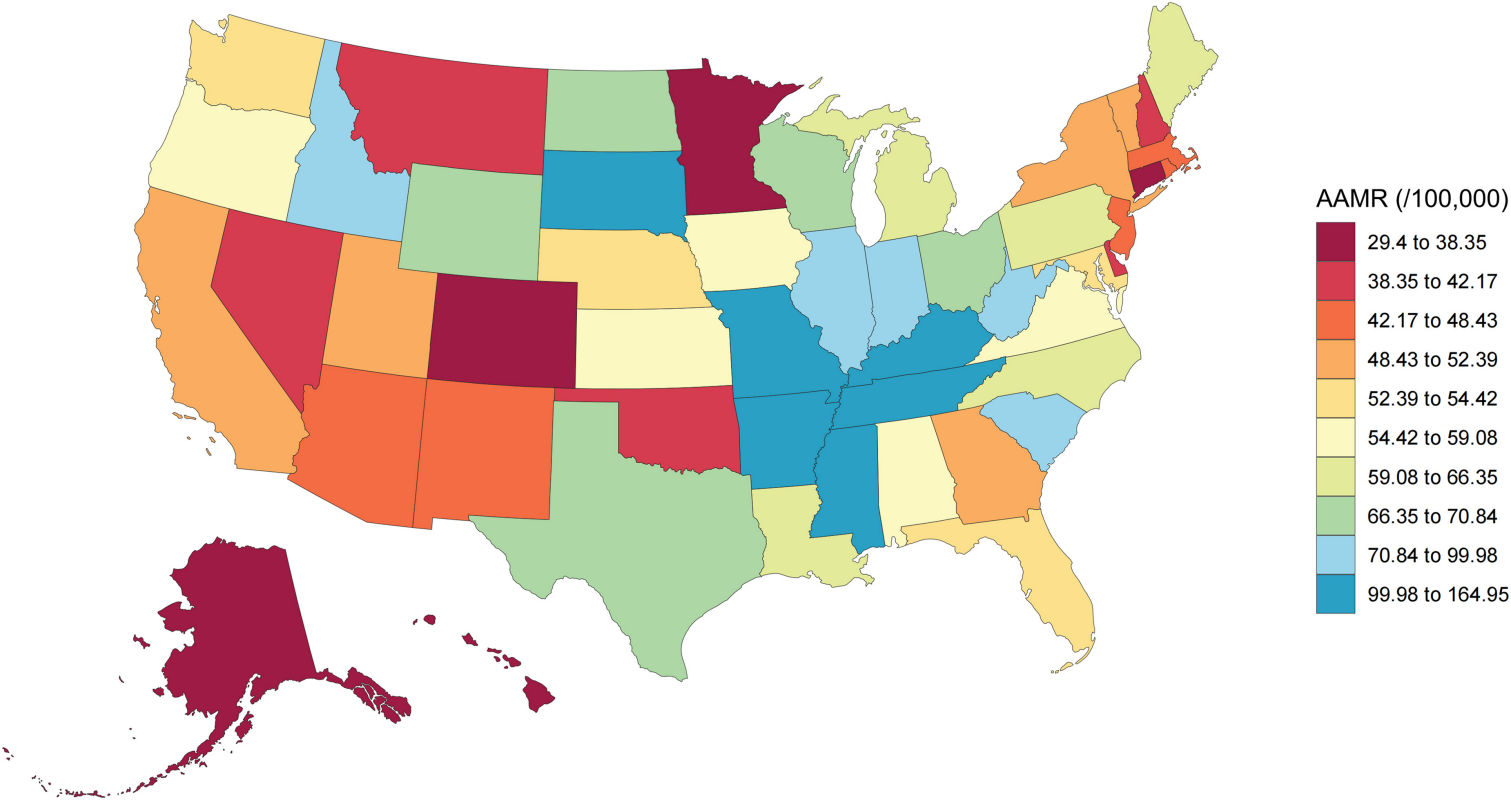
Geographic distribution of AMI-related age-adjusted mortality rates (AAMRs) across US states in 2023. This choropleth map illustrates the spatial heterogeneity of AMI-related mortality among adults aged ≥ 45 years. AAMRs ranged from 29.40 to 164.95 per 100,000, revealing a nearly six-fold difference between the highest and lowest-burden states. States in the South and parts of the Midwest exhibit disproportionately higher mortality rates compared to the Northeast and West. AAMR, Age-adjusted mortality rate.

Regional analysis revealed that the South consistently maintained the highest mortality burden, ending in 2023 with an AAMR of 68.99, while the Northeast achieved the most significant long-term improvement (AAPC: −5.39; Figure 3, Table 1, and Supplementary Table 3). The urban-rural divide was particularly stark. In 1999, rural areas already faced higher mortality than large metropolitan centers; by 2020, this gap had widened significantly. The AAMR in rural counties (116.41) was nearly double that of large metropolitan areas (59.44). Furthermore, large metropolitan areas showed a much faster rate of decline (AAPC: −5.58) compared to the slower progress observed in rural regions (AAPC: −3.92; Figure 4, Table 1, and Supplementary Table 4). At the state level, the magnitude of improvement varied more than three-fold (Supplementary files). States with the fastest declines included Delaware and Oklahoma whereas South Dakota and Mississippi experienced the slowest progress. By 2023, a nearly six-fold difference in mortality persisted between the highest and lowest-burden states (ranging from 29.40 to 164.95 per 100,000), highlighting profound spatial inequality across the United States (Figure 5).

## 4 Discussion

Based on national mortality registry data from CDC WONDER, this study provides a comprehensive longitudinal analysis of AMI-related AAMRs among US adults aged ≥ 45 years from 1999 through 2023. Our findings demonstrate that while AMI-related AAMRs declined significantly over the long term, this progress was interrupted by a phase-specific plateau during 2018–2021, followed by a renewed and accelerated decline through 2023. While this pattern aligns with prior US epidemiologic studies showing substantial but inconsistent improvements (13, 14), our study extends the current literature by revealing a post-pandemic shift toward a new mortality trajectory. A unique contribution of this analysis is the granular identification of persistent geographic heterogeneity, most notably a nearly six-fold difference in mortality rates across US states in 2023, which persists despite decades of clinical and technological advances. These results suggest that while nationwide outcomes have improved, the benefits of evidence-based interventions remain unevenly distributed across urban-rural, racial/ethnic, and regional levels.

From the perspective of clinical care evolution, the sustained long-term decline in AMI mortality observed in this study is underpinned by the cumulative impact of evidence-based medical interventions. Over the past two decades, guidelines for the management of ST-segment elevation myocardial infarction (STEMI) and non-ST-segment elevation acute coronary syndrome (NSTE-ACS) have shifted toward shortening symptom-to-reperfusion times, optimizing regional collaborative care networks, and standardizing emergency workflows (15, 16). These systemic improvements, complemented by updated chest pain evaluation protocols (17), have significantly reduced the risk of missed or delayed diagnoses. Notably, the core strategies regarding reperfusion timeliness, transfer pathways, and network-based care are highly consistent across both US and European clinical guidelines (18), suggesting a globally synchronized drive toward improving AMI outcomes. Furthermore, the transition toward primary percutaneous coronary intervention (PCI) as the central reperfusion modality, alongside hospital-level process restructuring-such as shortening door-to-balloon times-has enhanced key clinical outcomes (19, 20). Collectively, the long-term decline recorded in our study reflects the successful integration of technological advances, process optimization, and rigorous guideline implementation across the US healthcare system.

A key finding requiring particular attention is the plateau observed during 2018–2021. This phase change substantially overlaps with the COVID-19 pandemic, a period characterized by significant disruptions in healthcare accessibility and shifts in patient care-seeking behaviors. It is hypothesized that this plateau may be attributed, at least in part, to pandemic-related factors rather than a primary shift in underlying cardiovascular risk. Multicenter studies in the United States demonstrated a marked decline in STEMI catheterization laboratory activations during the early pandemic, suggesting that delayed care-seeking, inadequate prehospital recognition, or widespread healthcare avoidance potentially increased out-of-hospital mortality risk (21). Furthermore, the redistribution of hospital resources and strain on emergency medical systems during COVID-19 surges may have compromised the timeliness of reperfusion therapies (22, 23). While some studies suggest the decline in AMI presentations does not indicate a true reduction in disease burden, it is possible that some cases were “shifted” out of acute care datasets due to prehospital deaths or diagnostic prioritization shifts during the crisis. Therefore, the 2018–2021 trend pattern should be interpreted cautiously as a complex interplay between public health events, healthcare system capacity pressures, and altered patient behaviors.

From the perspective of shifting risk-factor profiles, the decelerating decline in AMI-related mortality may reflect a “countervailing effect” driven by an increasing metabolic burden. The long-term rise in the prevalence of obesity and diabetes among US adults is hypothesized to elevate both AMI incidence and mortality risk through pathways such as insulin resistance, systemic inflammation, and accelerated atherogenesis (24, 25). Simultaneously, recent evidence indicating stagnating or declining hypertension control rates suggests that real-world chronic disease management faces persistent challenges in medication adherence, healthcare accessibility, and continuity of care (26). Consequently, the observed plateau in AMI-related mortality during 2018–2021 is likely the multifaceted result of continued advancements in acute clinical care being increasingly offset by a worsening upstream risk-factor burden, a trend potentially exacerbated by the healthcare disruptions experienced during the COVID-19 pandemic.

The urban-rural and racial/ethnic disparities observed in this study are also highly informative. Regarding urban-rural differences, persistently higher AAMRs and slower declines in rural/county areas suggest that healthcare resource density, PCI-center accessibility, prehospital transport time, primary-level chronic disease management capacity, and socioeconomic factors may jointly shape AMI outcomes. The AHA scientific statement on social determinants of cardiovascular disease indicates that differences in education, income, health insurance, community environment, and structural resource allocation can affect cardiovascular outcomes through multiple mechanisms, including risk-factor exposure, care pathways, and treatment adherence (27). Regarding racial/ethnic differences, persistently higher AAMRs among non-Hispanic Black/African American individuals suggest that structural health inequities remain insufficiently addressed in the AMI-related mortality burden. In addition to social determinants, differences in symptom recognition, treatment delay, and care pathways may also contribute. The AHA scientific statement on AMI in women emphasizes differences across sexes in symptom profiles, pathophysiology, and care processes; this “heterogeneous care need” likewise suggests vigilance regarding unequal benefits from standardized pathways across subgroups (28). A JAMA study further confirmed complex interactions between AMI symptom presentation and in-hospital mortality risk across age and sex, providing a clinical basis for interpreting the differences observed in the age- and sex-stratified results of this study (29).

From the therapeutic evidence-chain perspective, the long-term decline in AMI-related mortality reflects the cumulative effects of multiple generations of evidence-based interventions. The ISIS-2 trial established the key role of aspirin (and its combination with streptokinase) in reducing early mortality risk in AMI and remains a major milestone in modern AMI pharmacotherapy (30). The SAVE trial further demonstrated that ACEI (captopril) use in patients with left ventricular dysfunction after AMI improves long-term mortality and cardiovascular outcomes, thereby promoting standardization of post-AMI secondary prevention (31). In antiplatelet therapy, the CURE trial established the clinical benefit of clopidogrel plus aspirin in patients with NSTE-ACS (32); the TRITON-TIMI 38 trial supported the use of a more potent P2Y12 inhibition strategy in appropriate patients to further reduce ischemic events (33); and the PLATO trial showed an outcome advantage of ticagrelor over clopidogrel in patients with ACS (34). In lipid management, the PROVE IT-TIMI 22 trial suggested that intensive statin therapy after ACS is superior to a moderate-intensity strategy (35); the IMPROVE-IT trial showed that adding ezetimibe on top of statin therapy can further reduce event risk (36); later, the FOURIER and ODYSSEY OUTCOMES trials extended evidence for PCSK9 inhibitors to lower residual-risk management (37, 38). These advances in pharmacotherapy, together with optimization of reperfusion strategies, are likely the core evidence base underlying the long-term decline in AMI-related mortality.

Meanwhile, further improvement in AMI-related mortality increasingly depends on continuum-of-care management rather than acute-phase treatment alone. The SPRINT trial showed that intensive blood pressure control can reduce major cardiovascular events and all-cause mortality, which has important implications for blood pressure targets in both primary and secondary prevention of AMI (39). Exercise-based cardiac rehabilitation can reduce cardiovascular mortality and rehospitalization risk in patients with coronary heart disease, but real-world participation rates and accessibility remain clearly inadequate (40). Systematic reviews show that smoking cessation in patients with coronary heart disease is significantly associated with lower mortality risk and is a cost-effective and feasible intervention (41). In addition, the PEGASUS-TIMI 54 trial suggested that, in patients with prior MI, long-term intensified antiplatelet therapy can further reduce ischemic events when based on strict population selection and bleeding-risk assessment, providing a basis for refined long-term management in high-risk patients (42). Therefore, the key to sustained future reductions in AMI-related mortality burden lies in organically integrating high-quality acute care with long-term risk-factor control, behavioral interventions, rehabilitation follow-up, and precision secondary prevention.

We further compared our findings with prior pre-pandemic and population-based studies to assess the consistency of our ICD-10 coding strategy (I21–I22) and trend estimates. A CDC WONDER-based analysis of AMI mortality among older US adults reported a substantial decline in AAMRs from 1999 to 2019 and an AAPC of approximately −4.96%, which is highly consistent with the overall AAPC of −4.94% observed in our study. Similarly, prior population-based and Medicare studies by Yeh et al. (13) and Krumholz et al. (14) documented substantial long-term improvements in AMI incidence, treatment, and outcomes before the pandemic, supporting the broader downward trend observed in our analysis. Regarding urban-rural disparities, our finding that rural areas had slower improvement than large metropolitan areas is consistent with prior CDC WONDER-based evidence showing persistent rural-urban inequalities in cardiovascular mortality (5). Racial/ethnic patterns, with non-Hispanic Black individuals having the highest AAMRs, were also generally consistent with previous population-level reports. Collectively, these comparisons support the validity of our ICD-10 coding strategy and indicate that our AAMRs, AAPCs, and temporal patterns are broadly concordant with pre-pandemic literature, while extending prior evidence through 2023.

This study has several limitations. First, while death certificate coding data are suitable for nationwide long-term trend surveillance, they may be subject to variations in cause-of-death reporting, changes in coding practices over time, and potential misclassification bias. Second, as a descriptive analysis utilizing the CDC WONDER platform, this study lacks clinical granularity regarding individual-level variables, such as disease severity, symptom-to-door duration, specific reperfusion modalities, and post-discharge medication adherence. Consequently, it is impossible to definitively distinguish whether the observed 2018–2021 plateau reflects a true shift in AMI incidence or a temporary alteration in care-seeking behaviors and diagnostic prioritization during the pandemic. Third, the reliance on aggregated mortality data precludes making definitive causal attributions regarding the observed temporal trends. Fourth, although sensitivity analyses using the Multiple Cause of Death (MCD) dataset (10) confirmed the robustness of the overall trend, the underlying mechanistic drivers behind the identified urban-rural and interstate disparities require further spatiotemporal analyses that integrate granular data on healthcare resource allocation, provider density, and structural social determinants of health.

Overall, this study suggests that despite the long-term decline in AMI-related mortality in the United States, substantial structural disparities and phase-specific fluctuations persist. Future public health and clinical management priorities should shift from “broadly improving treatment capacity” toward “precisely identifying high-burden populations and narrowing health disparities,” including strengthening emergency care network coverage in rural areas, improving risk-factor control in high-risk populations, enhancing adherence to secondary prevention, and optimizing long-term rehabilitation management, thereby further reducing the AMI-related mortality burden beyond current gains.

## 5 Conclusion

From 1999 to 2023, AMI-related age-adjusted mortality rates among US adults aged ≥ 45 years declined significantly overall, but a plateau occurred during 2018–2021, followed by a renewed decline; persistent urban-rural, racial/ethnic, and interstate disparities were also observed. Prevention and treatment resources should be further focused on rural areas and high-burden states by strengthening primary prevention, expanding emergency care network coverage, and improving access to evidence-based treatment to reduce health disparities.

## Data Availability

Publicly available datasets were analyzed in this study. These data can be found in the CDC WONDER database.

## References

[1] Thygesen K Alpert JS Jaffe AS Chaitman BR Bax JJ Morrow DA et al. Fourth universal definition of myocardial infarction (2018). *Circulation.* (2018) 138:e618–51. 10.1161/CIR.0000000000000617 30571511

[2] Ford ES Ajani UA Croft JB Critchley JA Labarthe DR Kottke TE et al. Explaining the decrease in U.S. deaths from coronary disease, 1980-2000. *N Engl J Med.* (2007) 356:2388–98. 10.1056/NEJMsa053935 17554120

[3] Shah NS Lloyd-Jones DM O’Flaherty M Capewell S Kershaw KN Carnethon M et al. Trends in cardiometabolic mortality in the United States, 1999-2017. *JAMA.* (2019) 322:780–2. 10.1001/jama.2019.9161 31454032 PMC6714016

[4] Wilmot KA O’Flaherty M Capewell S Ford ES Vaccarino V. Coronary Heart disease mortality declines in the United States from 1979 through 2011: evidence for stagnation in young adults, especially women. *Circulation.* (2015) 132:997–1002. 10.1161/CIRCULATIONAHA.115.015293 26302759 PMC4828724

[5] Cross SH Mehra MR Bhatt DL Nasir K O’Donnell CJ Califf RM et al. Rural-Urban differences in cardiovascular mortality in the US, 1999-2017. *JAMA.* (2020) 323:1852–4. 10.1001/jama.2020.2047 32396176 PMC7218488

[6] Wadhera RK Shen C Gondi S Chen S Kazi DS Yeh RW. Cardiovascular deaths during the COVID-19 pandemic in the United States. *J Am Coll Cardiol.* (2021) 77:159–69. 10.1016/j.jacc.2020.10.055 33446309 PMC7800141

[7] Friede A O’Carroll PW Thralls RB Reid JA. CDC WONDER on the Web. *Proc AMIA Annu Fall Symp.* (1996):408–12. 8947698 PMC2232915

[8] Kim HJ Fay MP Feuer EJ Midthune DN. Permutation tests for joinpoint regression with applications to cancer rates. *Stat Med.* (2000) 19:335–51. 10.1002/(SICI)1097-0258(20000215)19:33.0.CO;2-Z 10649300

[9] Waqas SA Imran Z Ali D Abramov D Virani SS Ahmed R et al. CDC WONDER: Trends in acute myocardial infarction mortality in the United States From 1968 to 2021. *JACC Adv.* (2025) 4:102010. 10.1016/j.jacadv.2025.102010 40680493 PMC12296419

[10] Minhas AMK, Sperling LS Al-Kindi S Abramov D. Underlying and contributing causes of mortality from CDC WONDER—Insights for researchers. *Am Heart J Plus.* (2025) 50:100499. 10.1016/j.ahjo.2025.100499 39895921 PMC11782113

[11] Ullah I Farooqi HA Ahmad O Irfan M Khan E Khan OA et al. Trends of acute myocardial infarction-related deaths in US patients from 1999 to 2020. *Arch Med Sci Atheroscler Dis*. (2024) 9:251–58. 10.5114/amsad/199656 40007989 PMC11851306

[12] Ingram DD Franco SJ 2013 NCHS urban-rural classification scheme for counties. *Vital Health Stat 2.* (2014) 166:1–73. 24776070

[13] Yeh RW Sidney S Chandra M Sorel M Selby JV Go AS. Population trends in the incidence and outcomes of acute myocardial infarction. *N Engl J Med.* (2010) 362:2155–65. 10.1056/NEJMoa0908610 20558366

[14] Krumholz HM Normand ST Wang Y. Twenty-Year trends in outcomes for older adults with acute myocardial infarction in the United States. *JAMA Netw Open.* (2019) 2:e191938. 10.1001/jamanetworkopen.2019.1938 30874787 PMC6484647

[15] O’Gara PT Kushner FG Ascheim DD Casey DE Jr. Chung MK de Lemos JA et al. 2013 ACCF/AHA guideline for the management of st-elevation myocardial infarction. *Circulation.* (2013) 127:e362–425. 10.1161/CIR.0b013e3182742cf6 23247304

[16] Amsterdam EA Wenger NK Brindis RG Casey DE Ganiats TG Holmes DR et al. 2014 AHA/ACC guideline for the management of patients with Non–ST-Elevation acute coronary syndromes. *Circulation.* (2014) 130:e344–426. 10.1161/CIR.0000000000000134 25249585

[17] Gulati M Levy PD Mukherjee D Amsterdam E Bhatt DL Blankstein R et al. 2021 AHA/ACC/ASE/CHEST/SAEM/SCCT/SCMR guideline for the evaluation and diagnosis of chest pain: executive summary. *Circulation.* (2021) 144:e368–454. 10.1161/CIR.0000000000001030 34709928

[18] Ibanez B James S Agewall S Antunes MJ Bucciarelli-Ducci C Bueno H et al. 2017 ESC guidelines for the management of acute myocardial infarction in patients presenting with ST-segment elevation. *Eur Heart J.* (2018) 39:119–77. 10.1093/eurheartj/ehx393 28886621

[19] Keeley EC Boura JA Grines CL. Primary angioplasty versus intravenous thrombolytic therapy for acute myocardial infarction: a quantitative review of 23 randomised trials. *Lancet.* (2003) 361:13–20. 10.1016/S0140-6736(03)12113-7 12517460

[20] Bradley EH Herrin J Wang Y Barton BA Webster TR Mattera JA et al. Strategies for reducing the Door-to-Balloon time in acute myocardial infarction. *N Engl J Med.* (2006) 355:10. 10.1056/NEJMsa063117 17101617

[21] Garcia S Albaghdadi MS Meraj PM Schmidt C Garberich R Jaffer FA et al. Reduction in ST-Segment elevation cardiac catheterization laboratory activations in the United States during COVID-19 pandemic. *J Am Coll Cardiol.* (2020) 75:2871–2. 10.1016/j.jacc.2020.04.011 32283124 PMC7151384

[22] Solomon MD McNulty EJ Rana JS Leong TK Lee C Sung S-H et al. The Covid-19 pandemic and the incidence of acute myocardial infarction. *N Engl J Med.* (2020) 383:10. 10.1056/NEJMc2015630 32427432

[23] Mafham MM Spata E Goldacre R Gair D Curnow P Bray M et al. COVID-19 pandemic and admission rates for and management of acute coronary syndromes in england. *Lancet.* (2020) 396:10. 10.1016/S0140-6736(20)31356-8 32679111 PMC7429983

[24] Hales CM Fryar CD Carroll MD Freedman DS Ogden CL. Trends in obesity and severe obesity prevalence in US youth and adults by sex and age, 2007-2008 to 2015-2016. *JAMA.* (2018) 319:10. 10.1001/jama.2018.3060 29570750 PMC5876828

[25] Menke A Casagrande S Geiss L Cowie CC. Prevalence of and trends in diabetes among adults in the United States, 1988-2012. *JAMA.* (2015) 314:1021–9. 10.1001/jama.2015.10029 26348752

[26] Muntner P Hardy ST Fine LJ Jaeger BC Wozniak G Levitan EB et al. Trends in blood pressure control among US adults with hypertension, 1999-2000 to 2017-2018. *JAMA.* (2020) 324:1190–200. 10.1001/jama.2020.14545 32902588 PMC7489367

[27] Havranek EP Mujahid MS Barr DA Blair IV Cohen MS Cruz-Flores S et al. Social determinants of risk and outcomes for cardiovascular disease: a scientific statement from the American Heart Association. *Circulation.* (2015) 132:873–98. 10.1161/CIR.0000000000000228 26240271

[28] Mehta LS Beckie TM DeVon HA Grines CL Krumholz HM Johnson MN et al. Acute myocardial infarction in women: a scientific statement from the American Heart Association. *Circulation.* (2016) 133:916–47. 10.1161/CIR.0000000000000351 26811316

[29] Canto JG Rogers WJ Goldberg RJ Peterson, Wenger NK Vaccarino V et al. Association of age and sex with myocardial infarction symptom presentation and in-hospital mortality. *JAMA.* (2012) 307:813–22. 10.1001/jama.2012.199 22357832 PMC4494682

[30] ISIS-2 (Second International Study of Infarct Survival) Collaborative Group Randomised trial of intravenous streptokinase, oral aspirin, both, or neither among 17,187 cases of suspected acute myocardial infarction: ISIS-2. *Lancet.* (1988) 2:349–60. 10.1016/S0140-6736(88)92833-4 2899772

[31] Pfeffer MA Braunwald E Moye LA Basta L Brown EJ Jr. Cuddy TE et al. Effect of captopril on mortality and morbidity in patients with left ventricular dysfunction after myocardial infarction: results of the Survival and Ventricular Enlargement Trial. *N Engl J Med.* (1992) 327:669–77. 10.1056/NEJM199209033271001 1386652

[32] Yusuf S Zhao F Mehta SR Chrolavicius S Tognoni G Fox KAA. Effects of clopidogrel in addition to aspirin in patients with acute coronary syndromes without ST-segment elevation. *N Engl J Med.* (2001) 345:494–502. 10.1056/NEJMoa010746 11519503

[33] Wiviott SD Braunwald E McCabe CH Montalescot G Ruzyllo W Gottlieb S et al. Prasugrel versus clopidogrel in patients with acute coronary syndromes. *N Engl J Med.* (2007) 357:2001–15. 10.1056/NEJMoa0706482 17982182

[34] Wallentin L Becker RC Budaj A Cannon CP Emanuelsson H Held C et al. Ticagrelor versus clopidogrel in patients with acute coronary syndromes. *N Engl J Med.* (2009) 361:1045–57. 10.1056/NEJMoa0904327 19717846

[35] Cannon CP Braunwald E McCabe CH Rader DJ Rouleau JL Belder R et al. Intensive versus moderate lipid lowering with statins after acute coronary syndromes. *N Engl J Med.* (2004) 350:1495–504. 10.1056/NEJMoa040583 15007110

[36] Cannon CP Blazing MA Giugliano RP McCagg A White JA Theroux P et al. Ezetimibe added to statin therapy after acute coronary syndromes. *N Engl J Med.* (2015) 372:2387–97. 10.1056/NEJMoa1410489 26039521

[37] Sabatine MS Giugliano RP Keech AC Honarpour N Wiviott SD Murphy SA et al. Evolocumab and clinical outcomes in patients with cardiovascular disease. *N Engl J Med.* (2017) 376:1713–22. 10.1056/NEJMoa1615664 28304224

[38] Schwartz GG Steg PG Szarek M Bhatt DL Bittner VA Diaz R et al. Alirocumab and cardiovascular outcomes after acute coronary syndrome. *N Engl J Med.* (2018) 379:2097–107. 10.1056/NEJMoa1801174 30403574

[39] Sprint Research Group, Wright JT Jr. Williamson JD Whelton PK Snyder JK Sink KM et al. A randomized trial of intensive versus standard blood-pressure control. *N Engl J Med.* (2015) 373:2103–16. 10.1056/NEJMoa1511939 26551272 PMC4689591

[40] Anderson L Thompson DR Oldridge N Zwisler AD Rees K Martin N et al. Exercise-based cardiac rehabilitation for coronary heart disease. *Cochrane Database Syst Rev.* (2016) 1:CD001800. 10.1002/14651858.CD001800.pub3 26730878 PMC6491180

[41] Critchley JA Capewell S. Mortality risk reduction associated with smoking cessation in patients with coronary heart disease: a systematic review. *JAMA.* (2003) 290:86–97. 10.1001/jama.290.1.86 12837716

[42] Bonaca MP Bhatt DL Cohen M Steg PG Storey RF Jensen EC et al. Long-term use of ticagrelor in patients with prior myocardial infarction. *N Engl J Med.* (2015) 372:1791–800. 10.1056/NEJMoa1500857 25773268

