# Supplementary material for: Trends in acute myocardial infarction mortality in the United States, 1999–2023
Source: Front Med (Lausanne). 2026 May 19;13:1817513. doi: 10.3389/fmed.2026.1817513 (PMC13226551; doi:10.3389/fmed.2026.1817513)

**Supplementary Table 1.** Acute myocardial infarction –related age-adjusted mortality rates per 100,000, stratified by sex in adults aged 45 years and older in the United States, 1999 to 2023.

| **Year** | **Age-Adjusted Mortality Rate (95% CI)** | | |
| --- | --- | --- | --- |
|  | **Overall** | **Male** | **Female** |
| 1999 | 206.12 (205.21-207.03) | 269.92 (268.22-271.62) | 159.75 (158.73-160.78) |
| 2000 | 196.82 (195.94-197.71) | 256.36 (254.72-258.00) | 153.39 (152.40-154.39) |
| 2001 | 184.93 (184.08-185.79) | 239.46 (237.89-241.03) | 144.91 (143.94-145.87) |
| 2002 | 176.80 (175.97-177.62) | 229.51 (227.99-231.03) | 138.01 (137.07-138.94) |
| 2003 | 164.75 (163.96-165.54) | 213.20 (211.75-214.65) | 128.48 (127.59-129.38) |
| 2004 | 148.96 (148.21-149.70) | 192.71 (191.36-194.07) | 115.98 (115.13-116.82) |
| 2005 | 140.43 (139.71-141.15) | 181.28 (179.98-182.58) | 109.41 (108.60-110.23) |
| 2006 | 128.48 (127.80-129.16) | 166.85 (165.63-168.08) | 99.04 (98.27-99.81) |
| 2007 | 118.17 (117.53-118.82) | 153.08 (151.92-154.24) | 91.12 (90.38-91.85) |
| 2008 | 116.62 (115.99-117.26) | 151.11 (149.97-152.24) | 89.81 (89.09-90.53) |
| 2009 | 106.91 (106.31-107.51) | 139.53 (138.46-140.61) | 81.47 (80.79-82.16) |
| 2010 | 102.13 (101.55-102.72) | 133.67 (132.63-134.72) | 77.35 (76.69-78.01) |
| 2011 | 97.44 (96.88-98.00) | 127.87 (126.87-128.87) | 73.34 (72.70-73.98) |
| 2012 | 93.46 (92.92-94.01) | 122.82 (121.86-123.79) | 70.05 (69.43-70.67) |
| 2013 | 90.45 (89.92-90.98) | 119.19 (118.25-120.13) | 67.40 (66.80-68.00) |
| 2014 | 86.32 (85.80-86.83) | 114.18 (113.27-115.08) | 63.85 (63.28-64.43) |
| 2015 | 84.31 (83.81-84.81) | 111.23 (110.35-112.11) | 62.49 (61.92-63.05) |
| 2016 | 81.02 (80.54-81.51) | 107.61 (106.75-108.46) | 59.35 (58.80-59.90) |
| 2017 | 78.10 (77.63-78.57) | 104.31 (103.48-105.14) | 56.71 (56.18-57.24) |
| 2018 | 75.26 (74.80-75.72) | 101.06 (100.25-101.87) | 54.03 (53.52-54.55) |
| 2019 | 70.91 (70.47-71.35) | 95.29 (94.51-96.06) | 50.84 (50.35-51.33) |
| 2020 | 72.81 (72.37-73.25) | 98.01 (97.23-98.79) | 51.92 (51.42-52.41) |
| 2021 | 74.23 (73.78-74.68) | 99.60 (98.81-100.39) | 53.08 (52.57-53.59) |
| 2022 | 67.93 (67.50-68.35) | 92.18 (91.43-92.93) | 47.92 (47.44-48.39) |
| 2023 | 60.71 (60.31-61.11) | 81.86 (81.16-82.56) | 43.12 (42.67-43.57) |

**Supplementary Table 2.** Acute myocardial infarction –related age-adjusted mortality rates per 100,000, stratified by race in adults aged 45 years and older in the United States, 1999 to 2023.

| **Year** | **Age-Adjusted Mortality Rate (95% CI)** | | |
| --- | --- | --- | --- |
|  | **NH Black or African American** | **NH White** | **Hispanic or Latino** |
| 1999 | 235.65 (232.22-239.08) | 207.72 (206.72-208.72) | 162.46 (158.51-166.42) |
| 2000 | 229.81 (226.44-233.18) | 198.10 (197.13-199.07) | 153.47 (149.72-157.22) |
| 2001 | 218.88 (215.62-222.14) | 185.76 (184.83-186.70) | 151.26 (147.64-154.88) |
| 2002 | 212.90 (209.70-216.10) | 177.22 (176.32-178.13) | 146.67 (143.20-150.14) |
| 2003 | 197.42 (194.36-200.48) | 165.60 (164.73-166.47) | 135.03 (131.78-138.28) |
| 2004 | 179.10 (176.22-181.99) | 149.89 (149.07-150.72) | 123.28 (120.25-126.30) |
| 2005 | 166.67 (163.92-169.41) | 141.25 (140.46-142.04) | 123.24 (120.31-126.18) |
| 2006 | 154.00 (151.40-156.61) | 129.40 (128.65-130.15) | 107.82 (105.15-110.50) |
| 2007 | 143.23 (140.75-145.71) | 119.22 (118.51-119.94) | 99.81 (97.31-102.31) |
| 2008 | 135.68 (133.30-138.06) | 118.70 (117.99-119.41) | 91.32 (89.00-93.63) |
| 2009 | 126.39 (124.12-128.65) | 108.73 (108.06-109.40) | 83.16 (81.02-85.31) |
| 2010 | 118.50 (116.33-120.67) | 103.98 (103.33-104.64) | 81.48 (79.41-83.56) |
| 2011 | 114.44 (112.35-116.54) | 99.77 (99.13-100.41) | 73.27 (71.38-75.16) |
| 2012 | 108.36 (106.36-110.36) | 95.86 (95.24-96.48) | 71.42 (69.61-73.22) |
| 2013 | 104.40 (102.47-106.33) | 92.82 (92.21-93.42) | 70.84 (69.09-72.58) |
| 2014 | 97.80 (95.96-99.63) | 89.18 (88.59-89.77) | 66.19 (64.56-67.82) |
| 2015 | 94.51 (92.75-96.28) | 87.18 (86.60-87.76) | 65.55 (63.98-67.12) |
| 2016 | 93.18 (91.46-94.91) | 83.66 (83.09-84.22) | 63.10 (61.60-64.61) |
| 2017 | 87.36 (85.72-89.00) | 81.11 (80.56-81.66) | 60.52 (59.09-61.96) |
| 2018 | 85.66 (84.06-87.26) | 78.02 (77.48-78.55) | 58.90 (57.52-60.28) |
| 2019 | 80.63 (79.10-82.16) | 73.71 (73.19-74.23) | 55.67 (54.36-56.98) |
| 2020 | 88.61 (87.03-90.19) | 74.39 (73.87-74.91) | 61.85 (60.50-63.20) |
| 2021 | 86.40 (84.82-87.98) | 77.64 (77.10-78.19) | 57.80 (56.50-59.11) |
| 2022 | 77.18 (75.72-78.65) | 71.61 (71.10-72.13) | 51.34 (50.15-52.54) |
| 2023 | 69.52 (68.14-70.90) | 64.25 (63.77-64.74) | 45.95 (44.84-47.07) |

**Supplementary Table 3.** Acute myocardial infarction –related age-adjusted mortality rates per 100,000, stratified by census region in adults aged 45 years and older in the United States, 1999 to 2023.

| **Year** | **Age-Adjusted Mortality Rate (95% CI)** | | | |
| --- | --- | --- | --- | --- |
|  | **Northeast** | **Midwest** | **South** | **West** |
| 1999 | 198.87 (196.92-200.81) | 220.53 (218.61-222.46) | 225.10 (223.49-226.72) | 162.62 (160.79-164.45) |
| 2000 | 189.56 (187.67-191.45) | 209.19 (207.32-211.06) | 217.57 (216.00-219.15) | 152.82 (151.06-154.58) |
| 2001 | 178.69 (176.87-180.51) | 193.52 (191.73-195.31) | 204.96 (203.44-206.48) | 146.24 (144.54-147.93) |
| 2002 | 170.84 (169.07-172.61) | 184.26 (182.52-185.99) | 195.49 (194.02-196.96) | 141.32 (139.67-142.97) |
| 2003 | 158.95 (157.25-160.64) | 171.03 (169.37-172.69) | 182.70 (181.29-184.10) | 132.03 (130.46-133.61) |
| 2004 | 142.96 (141.36-144.56) | 153.01 (151.45-154.57) | 166.28 (164.95-167.61) | 120.28 (118.79-121.76) |
| 2005 | 135.09 (133.55-136.64) | 145.50 (143.99-147.01) | 156.94 (155.66-158.22) | 111.44 (110.02-112.85) |
| 2006 | 121.15 (119.69-122.61) | 136.51 (135.06-137.96) | 142.01 (140.81-143.21) | 103.06 (101.72-104.40) |
| 2007 | 112.37 (110.98-113.76) | 123.31 (121.94-124.68) | 132.24 (131.10-133.38) | 93.69 (92.43-94.95) |
| 2008 | 109.97 (108.60-111.33) | 123.23 (121.88-124.59) | 129.64 (128.52-130.76) | 93.01 (91.77-94.25) |
| 2009 | 99.58 (98.29-100.88) | 112.82 (111.53-114.11) | 120.31 (119.24-121.37) | 84.21 (83.05-85.38) |
| 2010 | 94.41 (93.15-95.66) | 107.60 (106.35-108.86) | 115.32 (114.29-116.36) | 80.61 (79.49-81.74) |
| 2011 | 90.38 (89.17-91.60) | 106.05 (104.82-107.29) | 107.76 (106.78-108.74) | 76.76 (75.68-77.84) |
| 2012 | 85.38 (84.21-86.55) | 102.10 (100.90-103.30) | 104.00 (103.05-104.95) | 73.58 (72.54-74.63) |
| 2013 | 83.74 (82.58-84.89) | 97.77 (96.60-98.94) | 101.21 (100.29-102.14) | 70.35 (69.35-71.36) |
| 2014 | 79.04 (77.93-80.16) | 94.72 (93.59-95.86) | 96.67 (95.77-97.56) | 66.24 (65.28-67.20) |
| 2015 | 75.84 (74.76-76.93) | 90.87 (89.76-91.98) | 94.33 (93.46-95.20) | 67.60 (66.64-68.55) |
| 2016 | 72.57 (71.51-73.63) | 88.20 (87.12-89.29) | 90.12 (89.28-90.96) | 65.56 (64.63-66.48) |
| 2017 | 68.29 (67.28-69.30) | 84.73 (83.67-85.78) | 87.92 (87.10-88.74) | 63.02 (62.11-63.92) |
| 2018 | 66.11 (65.12-67.10) | 82.31 (81.28-83.34) | 85.17 (84.37-85.96) | 59.03 (58.17-59.89) |
| 2019 | 61.58 (60.63-62.53) | 77.90 (76.91-78.90) | 79.53 (78.77-80.29) | 57.07 (56.23-57.90) |
| 2020 | 62.33 (61.38-63.28) | 79.15 (78.15-80.15) | 82.45 (81.69-83.22) | 58.80 (57.96-59.64) |
| 2021 | 60.43 (59.49-61.37) | 80.91 (79.89-81.94) | 85.02 (84.23-85.81) | 61.07 (60.20-61.94) |
| 2022 | 55.11 (54.23-56.00) | 75.35 (74.38-76.31) | 76.78 (76.05-77.51) | 56.28 (55.46-57.09) |
| 2023 | 49.93 (49.10-50.77) | 66.89 (65.97-67.80) | 68.99 (68.30-69.67) | 49.81 (49.05-50.57) |

**Supplementary Table 4.** Acute myocardial infarction –related age-adjusted mortality rates per 100,000, stratified by urbanization in adults aged 45 years and older in the United States, 1999 to 2020.

| **Year** | **Age-Adjusted Mortality Rate (95% CI)** | | |
| --- | --- | --- | --- |
|  | **Large Metropolitan** | **Medium/Small Metropolitan** | **Rural/Counties** |
| 1999 | 192.21 (190.97-193.46) | 197.88 (196.26-199.50) | 258.25 (255.90-260.60) |
| 2000 | 181.86 (180.65-183.06) | 189.60 (188.03-191.18) | 249.97 (247.67-252.27) |
| 2001 | 171.90 (170.74-173.05) | 178.52 (177.01-180.03) | 232.13 (229.92-234.33) |
| 2002 | 163.08 (161.96-164.20) | 170.24 (168.78-171.71) | 226.46 (224.29-228.62) |
| 2003 | 151.33 (150.26-152.40) | 159.63 (158.23-161.03) | 211.62 (209.53-213.70) |
| 2004 | 136.20 (135.20-137.21) | 145.04 (143.71-146.36) | 192.05 (190.08-194.02) |
| 2005 | 126.77 (125.81-127.73) | 136.88 (135.60-138.15) | 185.68 (183.75-187.61) |
| 2006 | 115.88 (114.98-116.79) | 123.86 (122.66-125.05) | 172.81 (170.97-174.66) |
| 2007 | 105.00 (104.14-105.85) | 115.92 (114.77-117.06) | 160.34 (158.58-162.11) |
| 2008 | 102.82 (101.99-103.66) | 113.92 (112.80-115.04) | 161.51 (159.75-163.27) |
| 2009 | 93.26 (92.47-94.05) | 104.64 (103.57-105.70) | 151.10 (149.41-152.79) |
| 2010 | 88.28 (87.52-89.04) | 101.64 (100.60-102.68) | 144.05 (142.41-145.70) |
| 2011 | 83.27 (82.54-84.00) | 96.92 (95.92-97.92) | 141.12 (139.50-142.73) |
| 2012 | 78.43 (77.73-79.13) | 94.08 (93.11-95.06) | 138.34 (136.75-139.93) |
| 2013 | 75.70 (75.03-76.38) | 91.13 (90.18-92.08) | 135.00 (133.44-136.56) |
| 2014 | 71.64 (70.99-72.29) | 86.82 (85.90-87.74) | 131.29 (129.76-132.82) |
| 2015 | 69.89 (69.26-70.53) | 84.47 (83.58-85.37) | 129.75 (128.24-131.27) |
| 2016 | 67.24 (66.63-67.86) | 80.88 (80.01-81.75) | 125.61 (124.13-127.09) |
| 2017 | 64.11 (63.52-64.70) | 79.42 (78.57-80.28) | 121.37 (119.92-122.82) |
| 2018 | 61.87 (61.29-62.44) | 76.42 (75.59-77.25) | 117.15 (115.73-118.56) |
| 2019 | 57.82 (57.27-58.37) | 72.26 (71.46-73.06) | 111.66 (110.28-113.04) |
| 2020 | 59.44 (58.89-59.99) | 73.43 (72.64-74.23) | 116.41 (115.01-117.82) |

**Supplementary Figure 1.** Age-adjusted mortality rates (AAMRs) per 100,000 in adults aged 45 years and older in the United States from 1999 to 2023, with Acute myocardial infarction as a contributing cause of death and Cardiovascular disease as the underlying cause of death.


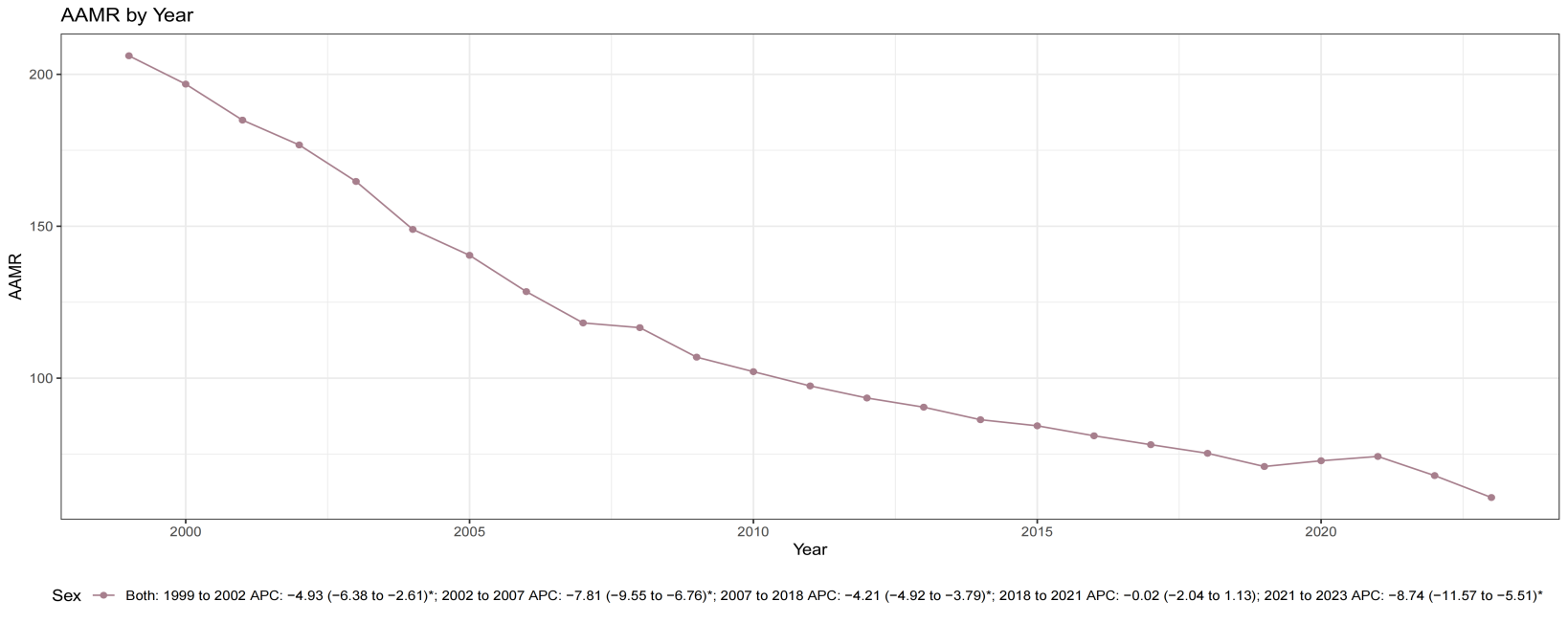

Supplement: Supplementary file 2 [file Table_1.docx]
